# The role of lactoferrin in bone remodeling: evaluation of its potential in targeted delivery and treatment of metabolic bone diseases and orthopedic conditions

**DOI:** 10.3389/fendo.2023.1218148

**Published:** 2023-08-23

**Authors:** Miao Tian, Ying-bo Han, Gui-yun Yang, Jin-long Li, Chang-sai Shi, Dan Tian

**Affiliations:** ^1^ Department of Gynecology and Obstetrics, The Second Hospital of Jilin University, Changchun, China; ^2^ Department of Gastrointestinal Surgery, The Second Hospital of Jilin University, Changchun, China; ^3^ Department of Operating Room, The Second Hospital of Jilin University, Changchun, China; ^4^ Department of Anesthesiology, The Second Hospital of Jilin University, Changchun, China

**Keywords:** lactoferrin, bone remodeling, signaling pathways, fracture repair, osteoporosis

## Abstract

Lactoferrin (Lf) is a multifunctional protein that is synthesized endogenously and has various biological roles including immunological regulation, antibacterial, antiviral, and anticancer properties. Recently, research has uncovered Lf’s critical functions in bone remodeling, where it regulates the function of osteoblasts, chondrocytes, osteoclasts, and mesenchymal stem cells. The signaling pathways involved in Lf’s signaling in osteoblasts include (low density lipoprotein receptor-related protein – 1 (LRP-1), transforming growth factor β (TGF-β), and insulin-like growth factor – 1 (IGF-1), which activate downstream pathways such as ERK, PI3K/Akt, and NF-κB. These pathways collectively stimulate osteoblast proliferation, differentiation, and mineralization while inhibiting osteoclast differentiation and activity. Additionally, Lf’s inhibitory effect on nuclear factor kappa B (NF-κB) suppresses the formation and activity of osteoclasts directly. Lf also promotes chondroprogenitor proliferation and differentiation to chondrocytes by activating the mitogen-activated protein kinase/extracellular signal-regulated kinase (MAPK/ERK) and phosphoinositide 3-kinase/protein kinase B(PI3K/Akt)signaling pathways while inhibiting the expression of matrix-degrading enzymes through the suppression of the NF-κB pathway. Lf’s ability to stimulate osteoblast and chondrocyte activity and inhibit osteoclast function accelerates fracture repair, as demonstrated by its effectiveness in animal models of critical-sized long bone defects. Moreover, studies have indicated that Lf can rescue dysregulated bone remodeling in osteoporotic conditions by stimulating bone formation and suppressing bone resorption. These beneficial effects of Lf on bone health have led to its exploration in nutraceutical and pharmaceutical applications. However, due to the large size of Lf, small bioactive peptides are preferred for pharmaceutical applications. These peptides have been shown to promote bone fracture repair and reverse osteoporosis in animal studies, indicating their potential as therapeutic agents for bone-related diseases. Nonetheless, the active concentration of Lf in serum may not be sufficient at the site requiring bone regeneration, necessitating the development of various delivery strategies to enhance Lf’s bioavailability and target its active concentration to the site requiring bone regeneration. This review provides a critical discussion of the issues mentioned above, providing insight into the roles of Lf in bone remodeling and the potential use of Lf as a therapeutic target for bone disorders.

## 1 Introduction

Several hormones and cytokines play a crucial role in bone metabolism; some of these have become therapies for osteoporosis. For example, estrogen has been used for many years in hormone replacement therapy to prevent and treat osteoporosis in postmenopausal women (1). Parathyroid hormone (PTH) and calcitonin are also approved for the treatment of osteoporosis and have been shown to increase bone density and reduce fracture risk (2). In addition to hormones, cytokines such as receptor activator of nuclear factor-kappa B ligand (RANKL) and sclerostin have also been identified as potential targets for osteoporosis therapy. Denosumab, a monoclonal antibody that targets RANKL, and romosozumab, a monoclonal antibody that inhibits sclerostin, have both been approved for the treatment of osteoporosis and have demonstrated significant benefits in increasing bone density and reducing fracture risk (3, 4). Endogenous factors naturally occur in the body and therefore have a lower risk of side effects and toxicity. Lactoferrin (Lf) is an endogenous protein in plasma that directly impacts bone cells and modulates bone metabolism (5), making it an attractive candidate for future research for positioning it as a therapeutic target for metabolic bone diseases.

Lf is an iron-binding glycoprotein required for several biological functions, including immune function, antimicrobial activity, and tissue repair. Human serum Lf range from 0.2 to 0.5μg/ml, mostly from neutrophils (6). There is no agreement among researchers on whether there are differences in plasma Lf levels between males and females (6).Lf is remarkably resistant to proteolytic degradation by enzymes such as trypsin, allowing it to be partially resistant to digestion in the gut, likely due to glycan-dependent resistance. The iron-saturated form, holo lactoferrin, is even more resistant to proteolysis than the iron-free form, apo lactoferrin, because the iron ion stabilizes its structure, making it less vulnerable to degradation (7). Resistance to proteolytic degradation enables Lf to be absorbed by the body, making it a significant nutrient for neonatal growth.

In recent years, there has been growing interest in the potential role of Lf in skeletal homeostasis, particularly in maintaining bone health and treating bone-related disorders such as osteoporosis. Studies have shown that Lf is expressed in bone cells, including osteoblasts and osteoclasts, and can modulate bone metabolism by promoting osteoblast differentiation and mineralization, inhibiting osteoclast activity, and regulating bone remodeling (5, 8, 9). The anti-inflammatory (10) and antioxidant (11) effects of Lf could contribute to its salutary effects on bone health. Given the beneficial effects of Lf on bone cells, it has therapeutic potential for treating metabolic bone disorders such as postmenopausal osteoporosis. Lf has also been the subject of extensive research in orthopedics. In recent years, there has been growing interest in using Lf-based therapies to treat various orthopedic conditions, such as fractures, osteoporosis, and implant-associated infections. A promising area of emerging research involves the targeted delivery of Lf to bones through drug delivery methods, in order to leverage its multiple beneficial effects on skeletal health. Expression of Lf receptors on the surface of osteoblasts, which are responsible for bone formation, allows specific targeting of this protein to the bone. Several preclinical and limited clinical research that would be discussed subsequently suggests that Lf has therapeutic promise in metabolic bone disorders and orthopedic applications. The focus of this narrative review is to examine and analyze the interplay between Lf and various cellular and molecular factors involved in maintaining bone health, as well as to assess the potential therapeutic benefits of using Lf-derived molecules for treating metabolic bone diseases and orthopedic conditions.

## 2 An overview of varied signaling by Lf

Lf has been shown to interact with various receptors and molecules, including CD14 (12), LDL receptor-related protein-1 (LRP-1/CD91) (13, 14), intelectin-1 (omentin-1) (15), Toll-like receptors 2 and 4 (TLR4) (16), cytokine receptor 4, and heparan sulfate proteoglycans (HSPGs) (17). CD14 is a glycosylphosphatidylinositol-anchored protein that acts as a co-receptor for toll-like receptor 4 (TLR4), a receptor recognizing bacterial lipopolysaccharides (LPS) (18). Lf has been shown to bind to CD14 and enhance the recognition of LPS by TLR4, leading to the activation of immune responses (19). LRP-1/CD91 is a multifunctional cell-surface receptor involved in various biological processes, such as endocytosis, cell signaling, and cell migration (20). Lf has been shown to bind to LRP-1/CD91 and regulate the internalization and degradation of the receptor (13). Intelectin-1 (omentin-1) is a lectin-like protein involved in various biological processes, including inflammation, cell adhesion, and angiogenesis. Lf has been shown to bind to intelectin-1 and regulate its biological functions (15). TLR4 is a receptor that recognizes various microbial components, such as LPS, and activates immune responses. Lf has been shown to activate TLR4 to enhance the production of pro-inflammatory cytokines (19). Cytokine receptor 4 (CXCR4) is a G protein-coupled receptor (GPCR) involved in various biological processes, such as cell migration, proliferation, and survival. Lf has been shown to bind to CXCR4 and regulate its signaling pathways (21). HSPGs are cell-surface and extracellular matrix macromolecules involved in various biological processes, such as cell adhesion, migration, and signaling. Lf has been shown to bind to HSPGs and regulate their biological functions, such as cell adhesion and migration (17). These interactions are critical in the innate immune system and other biological processes, such as inflammation, cell adhesion, migration, proliferation, and differentiation.

## 3 The regulation of bone cells by Lf and its associated signaling mechanisms

### 3.1 Mesenchymal stem cells (MSC)

In adult mammals, MSCs make up approximately 0.01% to 0.1% of the nucleated cells of bone marrow (22–24). Bone marrow MSCs can differentiate to osteoblasts, adipocytes and chondrocytes. In adult marrow, aging or altered metabolic conditions such as diabetes cause greater adipocyte differentiation over osteoblast differentiation leading to bone loss. Estrogen and PTH are two hormones that support increased osteogenic differentiation of bone marrow MSCs and concomitantly inhibit adipogenic differentiation thus favoring bone formation (25). In human bone marrow-derived MSCs, Lf treatment has been shown to suppress H_2_O_2_-derived reactive oxygen species (ROS) levels that likely inhibited senescence, and apoptosis of these cells. Moreover, the antiapoptotic effect of Lf in MSC involved inhibition of caspase-3 and activation of AKT activation (26). In MSCs obtained from adipose tissue, Lf showed mitogenic as well as pro-osteogenic effect demonstrated by the upregulation of Runx2 and ALP, and has the potential for bone tissue engineering applications (27). Indeed, incorporating Lf into biodegradable matrices containing hydroxyapatite, can enhance their osteogenic properties when applied to human MSCs (28, 29). However, there are no studies investigating whether Lf inhibits adipogenic differentiation of bone marrow-derived MSC which could have shed light on how Lf regulates the fate of MSCs, particularly their differentiation into osteoblasts and adipocytes.

### 3.2 Osteoblasts

Lf has been shown to regulate several molecular pathways in osteoblasts responsible for bone formation and remodeling. Several signaling pathways are involved in Lf’s actions in osteoblast proliferation, differentiation, and survival. The proximal signaling events identified for these actions include low-density lipoprotein receptor-related protein 1 (LRP1), IGF-1R, and TGFβ receptor pathways. LRP1 is a transmembrane receptor that can promote endocytosis of Lf (13). LRP1 can also produce cytoplasmic membrane-bound vesicles in osteoblasts, essential for the intracellular transport of proteins and other molecules. Lf has been shown to activate the extracellular signal-regulated kinase (ERK) pathway in osteoblasts through LRP1. Activation of the ERK pathway can stimulate osteoblast differentiation and bone formation, and Lf-mediated activation of this pathway may contribute to its osteogenic effects. On the other hand, Lf, through the PI3K/Akt pathway that is independent of LRP-1, inhibits osteoblast apoptosis has been reported (30).

Insulin-like growth factor 1-Insulin-like growth factor 1 receptor (IGF-1-IGF-1R)signaling plays a vital role in regulating bone metabolism, and a decline in IGF-1 has been implicated in age-related bone loss. Lf could address the decline in IGF-1 that occurs with aging by upregulating IGF-1 and IGF-1R in osteoblasts, which improved their viability under apoptotic stimulus (31). In senescent osteoblasts derived from SAMP6 mice (senescence-accelerated mouse-prone 6), an established model of accelerated aging that display several age-related phenotypes, including osteoporosis, sarcopenia, and cognitive decline, Lf enhanced the activity of the IGF1-Akt-mechanistic target of rapamycin (mTOR)signaling pathway. As a consequence of activating the IGF-1R-mediated osteogenic effect, Lf significantly attenuated the progression of osteoporosis due to senility (32). The suppression of senescent proteins, including p16 and p21, and oxidative injury through the upregulation of antioxidant enzyme activity through IGF-1R signaling likely attenuated the senescent-induced bone loss by Lf in SAMP6 model (32). Furthermore, Lf promoted the formation of osteoblasts from adipose tissue-derived stem cells (ADSCs) by activating the PI3K/AKT and IGF-R1 signaling pathways (33). Thus, it appears that to promote Lf’s osteogenic function, which includes osteoblast development from stem cells, osteoblast maturation, and osteoblast survival, the IGF-1-IGF-1R signaling is an effector arm. In bone marrow stromal cells (BMSCs), Lf and its digests activated the PI3K/AKT and ERK signaling pathways and promoted the expression of osteoblast-specific genes, such as runt-related transcription factor 2 (Runx2), alkaline phosphatase (ALP), and osteocalcin (OCN), while suppressing the expression of adipocyte-specific genes, such as peroxisome proliferator-activated receptor gamma(PPARγ)and fatty acid-binding protein 4 (FABP4). However, whether, IGF-1-IGF-1R signaling mediated the effect of Lf and its digests has not been studied (34).

Lf also activated the canonical TGF-β signaling pathway involving smad 2 via the TGF-β receptor II (TβRII), as demonstrated by the upregulation of osteogenic genes including Runx2, osterix, and collagen type I (35). Both canonical and noncanonical TGF-β signaling pathways were involved in the Lf-induced osteogenic activity of C3H10T1/2 MSCs. Lf treatment increased the phosphorylation of Smad2/3 and p38 MAP kinase, indicating the activation of canonical TGF-β signaling in the osteogenic differentiation of C3H10T1/2 cells. Lf also induced the phosphorylation of ERK1/2 in C3H10T1/2 cells, indicating the activation of noncanonical TGF-β signaling in the osteogenic differentiation of the cells (36).

**Figure 1:**
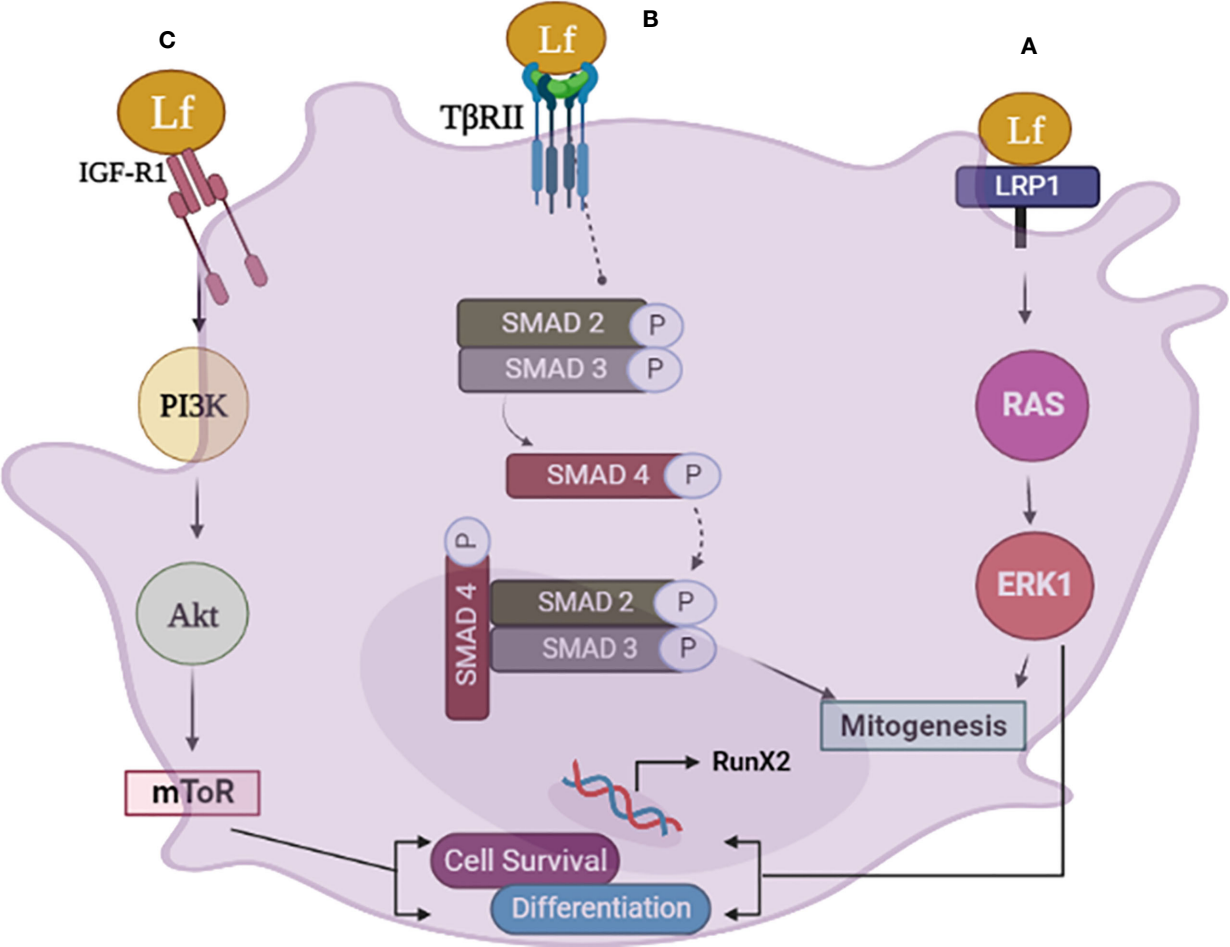
The schematic diagram illustrates the various pathways involved in lactoferrin (Lf) signaling in osteoblasts. **(A)** Lfsignaling via LRP1 in osteoblasts leads to mitogenesis and differentiation through the RAS-MAPK pathway. Lf binds to LRP1, triggering the activation of ERK, which promotes osteoblast differentiation and bone formation. The activation of ERK also induces mitogenesis in osteoblasts. **(B)** Lf can also signal through the TGFb receptor II (TβRII) in osteoblasts, activating smad 2, 3. This pathway results in the upregulation of osteogenic genes and promotes osteoblast differentiation. **(C)** Lfsignaling via the IGF-1 receptor in osteoblasts activates the PI3K/Akt and mTOR pathway, promoting osteoblast differentiation and survival independently of LRP1. This pathway promotes the survival of osteoblasts by inhibiting apoptosis. Image is made using the Biorender Software.

From the preceding discussion, it appears that Lfsignaling through LRP1, IGF-1R, and TGFβ receptor trigger a cascade of events in osteoblasts that result in the activation of several downstream pathways, including ERK1, PI3K, Akt. mTOR and smad2/3 that promote osteoblast growth, survival, and differentiation (for a schematic illustration, refer to **Figure 1**).

There are reports of additional signaling events elicited by Lf in osteoblasts besides the three receptors described above. For example, Lf stimulates the proliferation of osteoblasts through the upregulation of prostaglandin-endoperoxide synthase 2 (Ptgs2) (the enzyme encoding COX2) and NFATc1 activities. Inhibiting either COX2 or NFATc1 activity blocked the mitogenic effect of Lf in osteoblasts (37). Lf can also regulate gene expression by modulating long non-coding RNAs (lncRNAs). Knockdown of a specific lncRNA (RP11-509I15.3) that was upregulated by Lf treatment impaired osteogenic differentiation of rat BMSCs, suggesting that this lncRNA has roles in mediating the osteogenic effects of Lf (36).

In osteoblasts, Lf can inhibit the NF-κB signaling pathway, which is involved in the regulation of inflammatory responses, and is involved in osteoclast differentiation and bone resorption (38). Activation of NF-κB in osteoblasts results in the increased production of RANKL over OPG, which favors enhanced osteoclastogenesis. E2 negatively regulates RANKL, and its serum levels are increased after menopause (39). Consequently, denosumab, a human antibody against RANKL, is an approved therapy for postmenopausal osteoporosis (3). In the animal model of osteoporosis achieved by OVX, Lf suppressed the OVX-induced increases in RANKL: OPG ratio (38). Lf’s anti-oxidant/anti-inflammatory action appears to mediate this effect, although other regulatory mechanisms need to be investigated further.

### 3.3 Osteoclasts

Bone marrow cells are a heterogeneous population that includes osteoclast precursors, osteoblasts, and other cell types. Lipopolysaccharide (LPS), a component of the outer membrane of gram-negative bacteria, stimulates osteoclastogenesis (the formation of bone-destroying cells) by activating the RANKL signaling pathway in bone marrow cells. In mouse bone marrow cells, Lf inhibited LPS-induced osteoclastogenesis dose-dependently (9). When human peripheral CD14+ monocyte and macrophage cells were induced to differentiate into osteoclasts by a cocktail of macrophage colony-stimulating factor (M-CSF) and RANKL, Lf suppressed the expression of genes and proteins involved in osteoclast differentiation and activity such as TRAP and cathepsin K.

**Figure 2:**
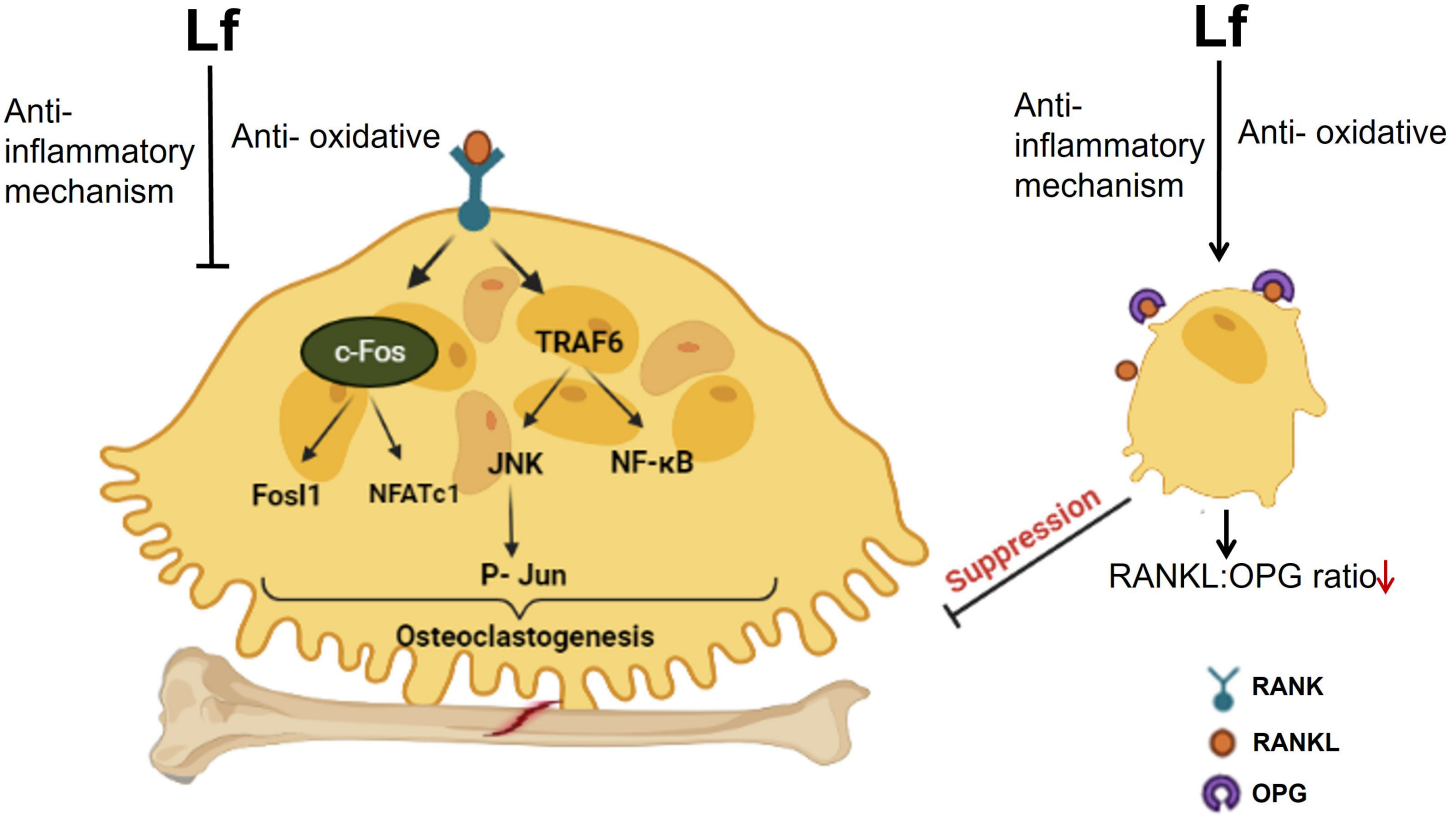
The schematic diagram illustrates the regulation of osteoclast function by Lf. Lf inhibits osteoclastogenesis in two ways. Firstly, it lowers the RANKL/OPG ratio by acting on osteoblastic cells. Secondly, it directly inhibits osteoclastogenesis through its anti-inflammatory effect, which prevents downstream signaling following RANKL binding to RANK. Moreover, Lf’s ability to scavenge free radicals also inhibits the generation of ROS involved in osteoclastogenesis. Image is made using the Biorender Software.

Overall, Lf regulates osteoclast function via two different mechanisms. Firstly, it lowers the RANKL/OPG ratio by influencing osteoblastic cells (9) and so suppressing osteoclastogenesis. Secondly, through its anti-inflammatory activity, Lf directly reduces osteoclastogenesis by blocking the downstream signaling that occurs when RANKL binds to RANK. Furthermore, by scavenging free radicals, Lf can limit the formation of ROS that are implicated in osteoclastogenesis (for a schematic illustration of osteoclast regulation by Lf, refer to **Figure 2**). The functional outcome of these suppressive effects was the inhibition of the resorption of bones by Lf ex vivo (40). Inhibition of osteoclastogenesis by Lf may have therapeutic potential for preventing bone loss associated with infectious diseases, periodontitis, and other inflammation-related diseases such as RA besides postmenopausal osteoporosis (41, 42).

### 3.4 Chondrocyte

Lf has a pro-survival effect in chondrocytes. Lf was found to prevent the programmed cell death of chondrocytes induced by interleukin-1 beta (IL-1β), a cytokine known to contribute to osteoarthritis (OA) development. Lf inhibited chondrocyte apoptosis by activating the protein kinase B (AKT1) pathway, which leads to the activation of the cAMP response element-binding protein 1 (CREB1) transcription factor. CREB1 plays a crucial role in regulating cell survival and has been shown to protect chondrocytes from apoptosis. When OA was induced in mice by destabilization of the medial meniscus (DMM) surgery in the knee joint, Lf significantly reduced cartilage degradation in the knee joints of the mice compared to the control group, as indicated by histological and immunohistochemical analyses (43).

Additionally, the Lf-treated mice showed significantly reduced chondrocyte apoptosis in the cartilage tissues of the knee joints, as indicated by TUNEL staining (43). Lf also protected chondrocytes from dexamethasone (Dex)-induced apoptosis by preventing the Dex-induced down-regulation of ERK1/2 and up-regulation of proteins involved in apoptosis, including FAS, FASL, and caspase 3 (44). By activating ERK1/2, Lf appears to preferentially increase the expression of BMP7, compared to BMP2 or BMP4 in chondrocytes (45). An increase in BMP7 expression in chondrocytes is generally considered beneficial, as it can enhance the ability of these cells to maintain and repair the cartilage matrix by promoting the proliferation, differentiation, and survival of these cells, as well as the synthesis of extracellular matrix proteins, such as collagen and proteoglycans. In the ATDC5 chondroprogenitor cell line, Lf inhibited their hypertrophic differentiation by inhibiting the expression of hypertrophic markers such as collagen X and ALP (46). Hypertrophic differentiation of chondrocytes leads to chondrocyte enlargement during the formation of bone, which is required for bone development. During bone development, chondrocytes undergo hypertrophic differentiation and contribute to the formation of mineralized bone tissue. However, excessive hypertrophic differentiation of chondrocytes can lead to cartilage breakdown and ultimately contribute to the progression of joint diseases such as OA.

## 4 Conservation of bone mass by Lf

When Lf was given to adult rats, it resulted in a significant increase in the rate at which minerals were deposited on the bone surface (mineral apposition rate), which is an indicator of osteoblast activity, and an increase in the overall amount of bone formed (cumulative bone formation rate) in skull bone (5). Lf supplementation was found to upregulate vitamin D receptor in both osteoblasts and kidneys, leading to improved BMD in both vitamin D deficient and vitamin D normal mice (47), highlighting its significance in maintaining appropriate vitamin D signaling necessary for optimal bone health. In SAMP6 mice, a model of aging and senescence, which are associated with decreased bone mass and increased risk of osteoporosis, Lf (2 g/kg/day) alleviated the adverse effects of age-related bone loss (32). Dietary supplementation or gavage administration of Lf prevented the loss of bone mass and strength in OVX mice and rats (38, 48–50). Femur is a critical bone for weight-bearing and movement, and the preservation of bone mass and strength in this bone by Lf is important for maintaining overall bone health and reducing the risk of fractures (49, 50). Thus it is conceivable that Lf may find therapeutic application in several disease conditions, including osteoporosis, chronic kidney disease, celiac disease, and vitamin D deficiency, where decreased bone formation rate is one of the reasons for bone loss over time.

Bone turnover markers (BTMs) are molecules released during the process of bone remodeling that include markers of bone resorption (β-CTx and NTx) and markers of bone formation (BALP, P1NP, osteocalcin, etc.) indicating the rate of bone turnover. BTMs are used as biomarkers in clinical trials for osteoporosis and related conditions, as they reflect changes in bone metabolism and can predict the risk of fractures. These markers are typically higher in OVX condition, where estrogen deficiency leads to increased bone resorption and decreased bone formation, resulting in osteoporosis. Higher levels of resorption markers and lower levels of formation markers are associated with bone loss and increased risk of fractures. Lf has been demonstrated to reduce the increases in bone resorption markers, such as CTX and NTx, induced by OVX. In addition, Lf increased markers of bone formation, such as osteocalcin and BSALP, compared to OVx animals (38, 49, 51). BTMs are useful in assessing the efficacy of drugs that aim to modify bone turnover, such as antiresorptive and anabolic agents. Given that Lf has been shown to affect BTMs in preclinical studies, including reducing bone resorption markers and increasing bone formation markers, it is a promising candidate for further investigation in clinical trials.

Consistent with the preclinical studies in OVX animals, where Lf reduced bone resorption markers, and increased bone formation markers, a study in healthy postmenopausal women reported that milk ribonuclease-enriched Lf supplementation resulted in a significant increase in osteocalcin and BSALP, and a decrease in β-CTx compared with the placebo control (52). Monitoring BTMs during clinical trials with Lf could provide insight into its mechanism of action and effectiveness in improving human bone health.

## 5 Effects of Lf in fracture healing

Fractures of long bones, especially large, comminuted, segmental, transverse, or open, are difficult to heal and have a high risk of non-union (failure to heal), especially in osteoporotic conditions (53).Nonunions require revision surgery that involves removing any hardware (such as screws or plates) used to stabilize the bone during the initial surgery and then using bone grafting to promote proper bone healing. When nonunions are suspected, BMPs (BMP-2/-7) are applied locally to the fracture site to stimulate the growth of new bone tissue and promote healing. However, BMPs (BMP-2/-7) are typically reserved for more complex or difficult-to-heal fractures and are not considered a first-line treatment for most fractures (54). Moreover, a high amount of BMP-2 in the graft can stimulate the local production of noggin, a protein that inhibits BMP-2 activity (55). This can lead to a negative feedback loop in which the efficacy of BMP-2 in promoting bone healing is diminished. As a result, bone growth promoters such as Lf could be evaluated for systemic delivery to reduce the rate of nonunions in large fractures.

The primary process involved in fracture healing is endochondral ossification. In this process, MSCs differentiate into chondrocytes that form a cartilage template, which is then mineralized. Blood vessels invade the calcified cartilage and bring osteoblasts that deposit new bone tissue, while osteoclasts break down and remodel the newly formed bone (56). Osteoporotic conditions can delay fracture healing by promoting excessive bone resorption and delaying the formation of nascent bone. This can lead to weaker callus formation and reduced bone strength at the fracture site, making it more prone to re-injury. Our preceding discussion described that Lf has salutary effects in osteoblasts, chondrocytes, and osteoclasts that support its use in nonunions by acting as a systemic bone growth promoter.

In the laboratory setting, the rabbit tibia is a commonly used model for studying long bone defects and fracture healing. The rabbit tibia offers several advantages as a model for studying bone healing, including its similar size and anatomy to human long bones and its weight-bearing. The unilateral tibial osteodistraction model is an animal model for studying bone regeneration and is often used to evaluate potential therapies for bone defects and fractures (57). In the osteodistraction model, a small cut (osteotomy) is made in the tibia, and an external fixator device is attached to the bone on either side of the osteotomy. The device is then slowly adjusted over time, causing the bone ends to gradually separate and new bone tissue to form in the gap between them. The osteodistraction technique can be used to study the effects of mechanical loading and other factors on bone formation and healing. Oral Lf was found to promote bone regeneration during distraction osteogenesis in rabbit tibia by increasing OPG to RANKL ratio, inhibiting the bone resorption rate (58). Another large bone defect model that is difficult to heal is a surgically created critical-sized defect. This defect is too large to heal on its own and is used in research to mimic open tibial fractures in humans that require orthopedic reconstructive procedures (59). By creating a critical-sized bone defect in the rabbit tibia, it is possible to study various interventions, such as bone grafting, growth factors, and tissue engineering, to promote bone regeneration and healing and to develop new treatments for orthopedic injuries and disorders in humans. An 18 amino acid peptide (RKVRGPPVSCIKRDSPIQ) from the N-terminus of the N-lobe of human Lf called LP2 stimulated bone regeneration in the critical-sized defect in rabbits by increasing the production of BMP-2 in osteoblasts. Additionally, the LP2 peptide had an anti-osteoclastogenic effect by enhancing the ratio of OPG to RANKL in osteoblasts (60). These data suggest that the upregulation of OPG is the critical mechanism underlying the healing of long bone fracture by Lf.

## 6 Designing therapeutic peptides from Lf FOR treating bone diseases

Lf is a large and multifunctional protein. Hence, small peptides made from Lf are useful for therapeutic purposes because they allow for more efficient delivery and targeting of specific biological functions. Furthermore, smaller peptides are less immunogenic than bigger proteins, lowering the risk of unfavorable immune responses and adverse effects. Smaller peptides are also more likely to penetrate tissues and reach their target cells or molecules, increasing their bioavailability and efficacy (61).

Positively charged amino acid segments, such as those containing arginine, lysine, and histidine, are often preferred for the design of bioactive peptides because they can interact with negatively charged molecules in biological systems. These positively charged amino acids, in particular, can generate electrostatic interactions with negatively charged cell membranes and other macromolecules, resulting in various biological effects. Moreover, positively charged amino acid segments can facilitate the uptake of peptides into cells, as some cellular uptake mechanisms are known to be selective for peptides with positively charged residues. This enhanced cellular uptake can increase the bioavailability and efficacy of peptides. Thus, the N-terminal region of Lf has become a focal point for designing peptides with potential therapeutic applications. Lactoferricin (62–64) and lactoferrampin (65, 66) deserve special mention because these have undergone extensive research for their anti-microbial effect. Both are cationic and α-helical peptides with a hydrophobic N-terminus and a hydrophilic C-terminus that are stable over a wide pH and temperature range (67).Lactoferricin has potent antimicrobial activity against a broad range of bacteria, fungi, and viruses, and lactoferrampin has broad-spectrum activity against bacteria, fungi, and protozoa (62–66). Given the anti-microbial property of Lf may be considered in the treatment of osteomyelitis, a bone infection commonly caused by Staphylococcus aureus. In this regard, a human Lf-derived peptide in both prophylactic and therapeutic modes significantly reduced severity of osteomyelitis in a rabbit model evident from improved microbiological, radiological and histological scores compared to the placebo group. Strikingly, the effect of the peptide was on a par with gentamicin (68, 69). The rabbit model of osteomyelitis mimics the progression of human disease because the long bones of rabbits are physiologically similar to humans - both species having Haversian remodeling. Hence, the findings of the reports showing the mitigation of osteomyelitis by Lf-derived peptide in the rabbit model of the disease holds potential translational value for human applications.

The anti-microbial mechanism of Lf-derived peptides could also be useful in affording protection against cartilage degradation. For example, lactoferricin inhibits the effects of IL-1 and fibroblast growth factor 2, which are known to cause cartilage degradation (70). Lactoferricin also induces the production of interleukin-11 (IL-11), an anti-inflammatory cytokine, which then activates the STAT3 signaling pathway to up-regulate the expression of TIMP-1 in human adult articular chondrocytes. The up-regulation of TIMP-1 expression by IL-11 may be a secondary cellular response after IL-11 induction by lactoferricin via the ERK-AP-1 axis (71). Together, these reports suggest that lactoferricin may have a dual mechanism of action in reducing inflammation in human articular cartilage by both inducing the production of anti-inflammatory cytokines and inhibiting the production of pro-inflammatory cytokines. These reports also underscore the potential of lactoferricin as a therapeutic agent for the treatment of inflammatory joint diseases such as OA. However, the effect of lactoferricin on metabolic bone diseases such as postmenopausal osteoporosis has not been investigated. As discussed in the preceding section, by suppressing inflammatory mediators including TNFα, IL-1β and IL-6, Lf/Lf-derived peptides also inhibit osteoclast formation and activity. In this regard, the effect of lactoferricin and other Lf-derived peptides on osteoclast formation and function, and inhibition of bone resorption *in vivo* has not been investigated.

Two Lf-derived peptides have been shown to have potential effects on osteoblast function. LFP-C, a 9-amino acid peptide was isolated from Lf hydrolysates by pepsin digestion enhanced osteoblast differentiation and mineralization and increased the expression of genes involved in bone formation (72). LP2 is another osteogenic peptide derived from human Lf. This synthetic peptide has a self-assembling property and assumes nanoglobular structures owing to which it spontaneously aggregate and form stable, spherical structures, without the need for external assembly factors or scaffolds. LP2, when systemically administered, demonstrated osteogenic and anti-resorptive effects in maintaining bone mass and strength in OVX rats by activating p38 MAPK and BMP-2 production and increasing OPG production, and in rabbits with a critical-sized defect in the tibia, it led to faster healing of the defect than control (60). For various Lf-derived peptides and their functions, refer to **Table 1**. Taken together, it appears that Lf-derived peptides hold great promise as potential therapeutic agents for the treatment of orthopedic and metabolic bone diseases.

**Table 1:** Lf-derived peptides with their activities.

Name	Sequence	Activity	Reference
Lactoferrin			
b-lactoferrin	FKSETKNLL	osteogenesis	(72)
h-lactoferrin	RKVRGPPVSCIKRDSPIQ	Osteogenesis	(60)
h-lactoferrin	GRRRRSVQWCA	Osteomyelitis	(68, 69)
Lactoferricin			
b-lactoferricin	FKCRR WQWRMKKLGAPSITCVRRAF	Anti-microbial	(63, 64)
b-lactoferricin	FKCRRWQWRMKKLG	Anti-microbial and antibiofilm	(73)
b-lactoferricin	FKCRRWQWRMKKLGAPSITCVRRAF	Anti-cancer	(74)
Lactoferrampin			
h-lactoferrampin	WNLLRQAQEKFGKDKSPK	Anti-viral	(65)
h-lactoferrampin	WNLLRQAQEKFGKDKSP	Anti-microbial	(66, 75)
d-lactoferrampin	WKLLSKAQEKFGKMKSR	Antimicrobial, candidacidal and anti-bacterial	(76, 77)

b, bovine; h, human; d, deer.

## 7 Delivery of Lf to the bone

Although Lf is present in serum, its active concentration may not be present at the site requiring bone regeneration. Bone regeneration is a complex process that requires the presence of various growth factors and biomolecules at the site of injury, and the levels of these factors can vary depending on the location and extent of the injury. Delivering Lf to the site of bone regeneration can ensure that it is present in sufficient quantities to promote bone growth and regeneration. Additionally, Lf delivery strategies can protect it from degradation and provide sustained release over time, further enhancing its effectiveness. Biocompatibility of Lf, i.e. non-toxicity to cells and tissue makes it an attractive target for delivery to the bone to promote bone growth, reduce inflammation, and prevent infections. Effective delivery of Lf to the site of bone regeneration has been achieved mostly through hydrogels, which protect it from degradation and enable sustained release over time.

Hydrogels are cross-linked polymer networks that can absorb large amounts of water while maintaining their three-dimensional structure and have a similar mechanical strength and elasticity to natural tissues (78). Lf can be added to the hydrogel during the synthesis process or can be incorporated after the hydrogel is formed. Once implanted at the site of bone regeneration, the hydrogel can slowly release Lf, providing sustained delivery over time. Additionally, the hydrogel can provide a matrix for cell attachment and proliferation, promoting bone growth and regeneration. Hydrogels can also be functionalized with specific chemical groups to enhance the delivery of Lf with other growth factors.

Gelatin hydrogels are hydrophilic and biodegradable, and they are commonly used in biomedical applications due to their biocompatibility and ability to mimic the extracellular matrix of natural tissues. When the release of Lf from a gelatin hydrogel was sustained for 28 days, it promoted the proliferation and differentiation of osteoblasts. In a rat femoral defect model, the Lf-releasing gelatin hydrogel resulted in bone regeneration. The newly formed bone showed good integration with the surrounding bone tissue and no signs of inflammation or necrosis (79).

Poloxamer hydrogels are a class of hydrogels made up of a triblock copolymer of poly(ethylene oxide)-poly(propylene oxide)-poly(ethylene oxide) that can exist as a liquid at low temperatures but form a gel at body temperature (80). However, poloxamer hydrogels are non-biodegradable and have a relatively low mechanical strength compared to gelatin hydrogels. Besides thermal reversibility, poloxamer hydrogels have good biocompatibility and low toxicity, making them suitable for various biomedical applications as injectable gel formulations. Poloxamer hydrogels loaded with Lf can sustain the release of Lf for up to 21 days, which promotes bone regeneration. This sustained release formulation of Lf enhanced the osteogenic differentiation of rat MSCs and improved mechanical strength compared to the non-loaded hydrogels. In a rat calvarial defect model, this formulation promoted bone regeneration and new bone formation, and the newly formed bone tissue showed no signs of inflammation or necrosis (81).

**Figure 3:**
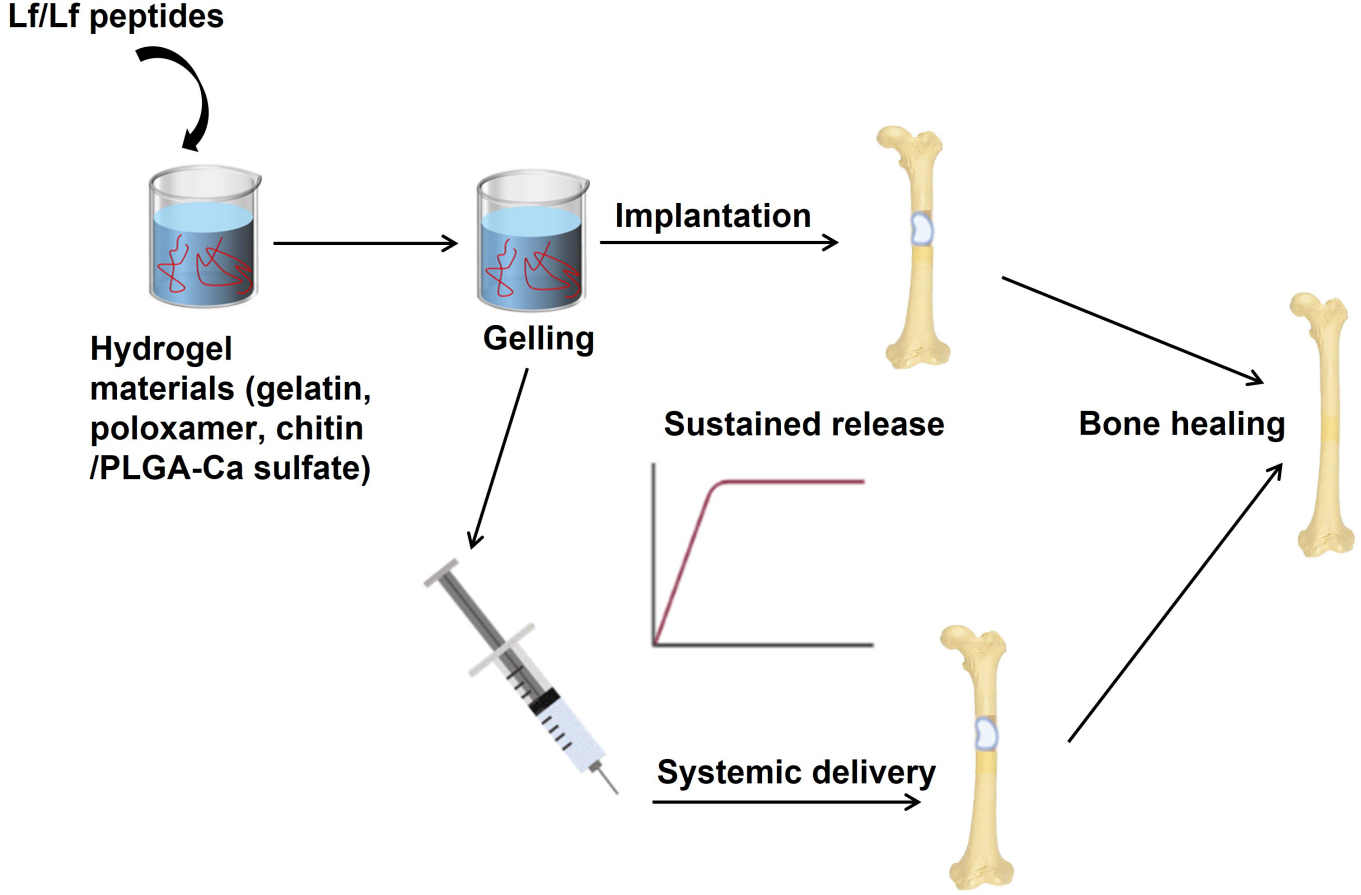
Strategies to improve the delivery of Lf or Lf-derived peptides to the bone. Hydrogels have been used in preclinical models of critical-sized bone defects (mimicking non-unions) as sustained-release formulations to deliver high amounts of Lf to bones. In these models, Lf has been used for local delivery of Lf as implants at the site of the bone defect. This approach ensures Lf’s sustained release, which can stimulate bone regeneration and repair. In addition, systemic delivery of Lf through hydrogels has also been explored. In this case, hydrogels containing Lf are injected into the bloodstream, allowing for the controlled release of Lf over time. These approaches have shown promising results in promoting bone regeneration in critical-sized bone defects. Image is made using the Biorender Software.

Chitin/PLGA-CaSO_4_ hydrogel has distinct advantages over other hydrogels, such as osteogenic and angiogenic activity, a porous structure, good biocompatibility, sol-gel transition at body temperature, and controlled release of bioactive molecules. The chitin/PLGA-CaSO_4_ hydrogel loaded with Lf and substance P significantly promoted bone regeneration and new bone formation in the calvarial bone defect model compared to the hydrogel alone or hydrogel loaded with Lf or substance P alone. As substance P has bone regenerative action and improves bone healing, it was included with Lf, which resulted in synergistic effects on bone regeneration and improved the therapeutic efficacy of the hydrogel. The combination of Lf and substance P enhanced the osteogenic and angiogenic activity of the hydrogel, as evidenced by increased expression of osteogenic and angiogenic markers *in vitro* and *in vivo* (82). The findings of this study suggest that combining Lf with other osteogenic agents, such as teriparatide, could potentially enhance the overall bone regeneration response. **Figure 3** describes various strategies to improve the delivery of Lf or Lf-derived peptides to the bone to accelerate critical-sized bone defects that are observed in comminuted fractures in humans.

## 8 Summary & future research

This review covered the roles of Lf bone remodeling and resorption, bone healing, and regeneration. LRP1, IGF-1R, and TGFβ receptor pathways have been identified as the proximal signaling events involved in the actions of Lf in osteoblasts. The downstream events of these proximal signaling pathways lead to the activation of ERK1/2, Akt, PI3K/Akt, MAPK, and SMAD pathways. These signaling pathways promote proliferation, migration, survival, differentiation, and extracellular matrix formation in osteoblasts, which are important for tissue repair and regeneration. Lf inhibits osteoclast differentiation and activity by suppressing NF-κB signaling, inducing OPG expression, and downregulating RANKL expression, thereby modulating key regulatory pathways involved in osteoclastogenesis and bone resorption. Lf also modulates matrix metalloproteinase activity, which is involved in bone remodeling. Lf’s actions on chondrocytes involve activating multiple signaling pathways, including Akt, CREB, ERK, and BMP7, which promote chondrogenesis, cell survival, and cartilage formation. Regulation of these signaling events in bone cells by Lf contributes to tissue repair and regeneration and inhibits bone loss in osteoporosis. By improving bone mineral density and reducing the risk of bone loss, Lf may help prevent fractures and other osteoporosis-related complications. In addition, further investigation is needed to elucidate the exact mechanisms of action of Lf on bone metabolism, including the role of Lf receptors in bone cells.

**Figure 4:**
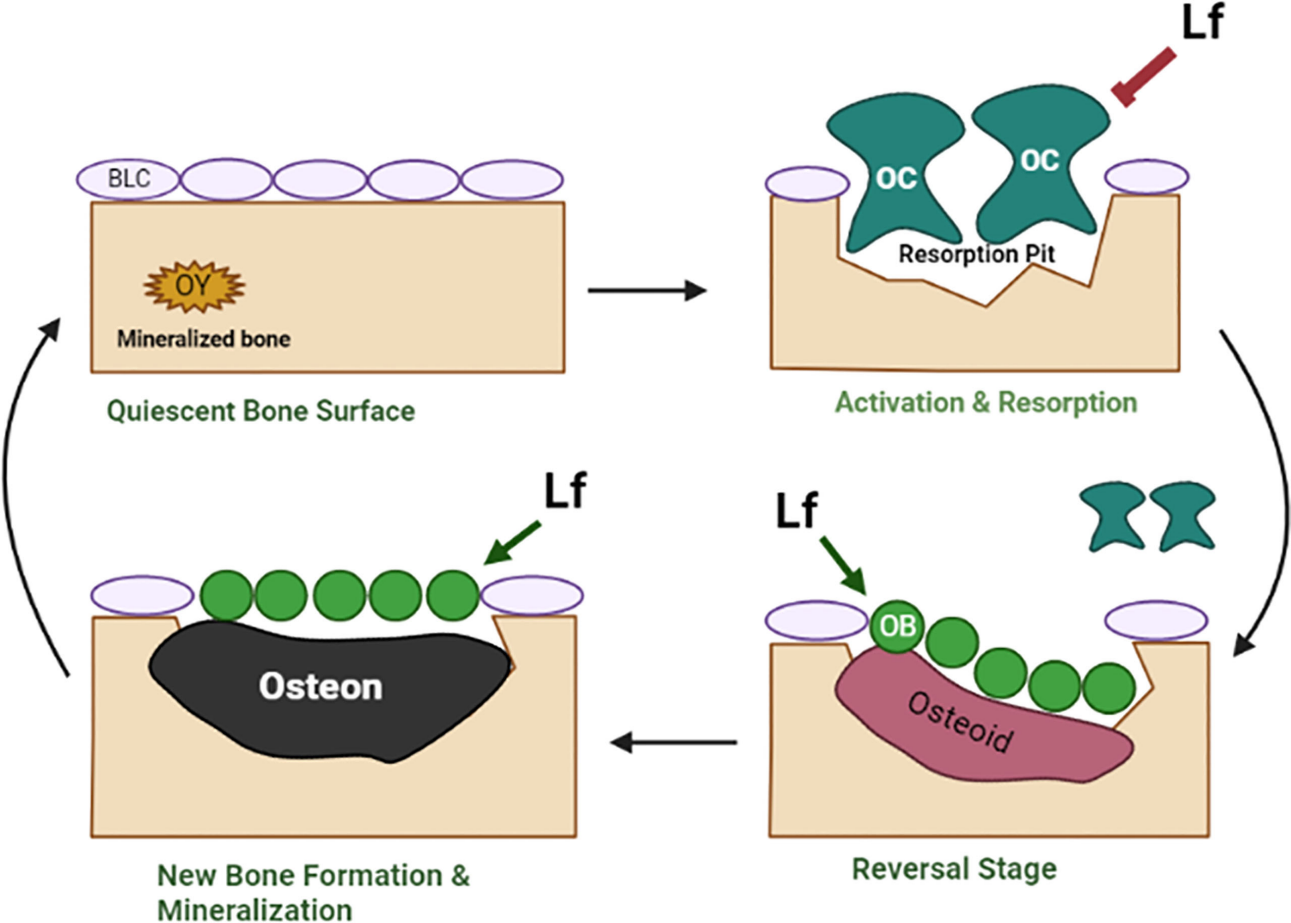
The schematic diagram shows the effects of Lf in bone remodeling. The bone remodeling cycle comprises several stages, including quiescence, activation & resorption, reversal, and formation. In the activation & resorption stage, osteoclasts are recruited to the bone surface and resorb the old bone. In the reversal stage, osteoblasts are recruited to the bone surface to begin the process of new bone formation. The final stage involves the production of new bone matrix by osteoblasts, which undergoes mineralization. In the normal bone remodeling cycle, there is a balance between bone resorption and bone formation such that the amount of bone that is resorbed is replaced by an equal amount of new bone formation, resulting in the maintenance of a constant bone mass. In osteoporosis, there is an imbalance between bone resorption and formation because osteoclast activity is increased. In contrast, osteoblast activity is decreased, resulting in decreased bone mass and an increased risk of fractures. Lf acts at the activation & resorption phases by the mechanisms described in **Figure 2** to inhibit bone resorption. Lf also acts at the reversal and formation stages to stimulate bone formation by the mechanisms described in **Figure 1**. By these mechanisms, Lf corrects the remodeling cycle and restores bone mass. Osteioid, unmineralized bone matrix; osteon, mineralized bone matrix. Image is made using the Biorender Software.

The limitations of current osteoporosis therapies are that they tend to have a one-sided approach that either inhibits bone resorption or stimulates bone formation. For example, bisphosphonates, which are one of the most commonly used osteoporosis drugs, inhibit bone resorption but do not stimulate bone formation. On the other hand, anabolic agents such as teriparatide and abaloparatide stimulate bone formation but have no effect on bone resorption. Therefore, there is a need for a therapy that can inhibit bone resorption as well as stimulate bone formation, providing a dual benefit for osteoporosis patients. Estrogen was once the only therapy that could inhibit bone resorption and stimulate bone formation. However, significant cancer and cardiovascular risks associated with estrogen use in postmenopausal women have resulted in its discontinuation. Therefore, there is currently no therapy that can provide the dual benefit of estrogen without the associated risks (for additional details, refer to **Figure 4**). Lf has the potential to fill the void left by estrogen as a therapy that can inhibit bone resorption and stimulate bone formation in osteoporosis patients. As an endogenously produced protein that has a good safety profile, low toxicity, and availability make it an attractive candidate for further investigation as a therapeutic agent for osteoporosis. However, further research is needed to fully understand its mechanisms of action and to determine its optimal dose and delivery route for therapeutic use.

Since osteogenic and chondrogenic peptides from Lf have already been found, a rational design strategy may be appropriate for developing more such peptides from Lf with improved function. By analyzing the amino acid sequences and structural features of existing osteogenic/chondrogenic peptides, key amino acid residues implicated in their biological activities can be identified and included in the design of novel osteogenic/chondrogenic peptides. Additionally, alanine scanning can be used to confirm the importance of specific amino acid residues identified through rational design and ensure that they are essential for the osteogenic/chondrogenic activity of the peptide. Combining these techniques can provide a more thorough understanding of the structure-function correlations of Lf-derived peptides and aid in generating novel osteogenic/chondrogenic peptides with increased potency and selectivity.

Hydrogels may enhance their therapeutic potential in bone-related applications by protecting Lf from degradation and enabling sustained release. Hence, hydrogels have been utilized as a drug delivery system and discussed here. However, further studies are needed to investigate the potential synergistic effects of combining Lf with other osteogenic agents in these hydrogels. There is also a need to develop hydrogels that mimic the complex mechanical properties of natural bone tissue and that can degrade over time and be replaced by new bone tissue. pH is an important factor during bone remodeling and the formulation of hydrogels because it can affect the solubility, stability, and bioactivity of biomolecules such as Lf. In bone regeneration, the pH of the local environment can affect the activity of bone cells. For example, a slightly acidic environment (pH 6.8-7.2) is beneficial for osteoclast activity required for initiating remodeling, while a slightly alkaline environment (pH 7.4-7.8) is beneficial for osteoblast activity. Therefore, enhancing the efficiency of Lf administration for bone regeneration may require regulating the pH of the local environment and the hydrogel formulation. This can be accomplished by using pH-sensitive hydrogels or by incorporating pH-modulating agents into the hydrogel formulation. Other approaches to more efficiently targeting Lf to bones may include encapsulating it in liposomes or polymeric nanoparticles and functionalizing the nanoparticles with bone-targeting molecules such as bisphosphonates, or conjugating Lf with bone-targeting peptides derived from osteocalcin or bone sialoprotein.

Future research areas for Lf and bone include determining its optimal dose and delivery routes for bone regeneration and osteoporosis. In addition, further investigation is needed to elucidate the exact mechanisms of action of Lf on bone metabolism, including the role of Lf receptors in bone cells. Clinical trials are also necessary to evaluate the safety and efficacy of Lf as an osteoporosis therapy in humans and to investigate its long-term effects on bone density and fracture risk. Additionally, considering its osteogenic and anti-resorptive effects, there is potential use for Lf in combination with osteogenic anti-osteoporosis drugs including teriparatide, abaloparatide or romosozumab, whether in the form of intact Lf, enzymatically digested Lf or a bioactive peptide such as LP2. Combining Lf or suitable Lf-derived peptide with any of the osteogenic drugs could potentially have a synergistic effect in the treatment of osteoporosis. Because Lf or the proteolytic digests have neutraceutical use, it could be conveniently assessed in clinical trials via oral administration. For Lf-derived peptides, however, given their parenteral route of administration, regulatory studies to assess safety, efficacy and optimal dosage are required before their use in humans. Finally, Lf may have therapeutic applications in other bone diseases, such as OA and periodontitis, and further research is needed to explore its potential in these conditions.

Overall, the research on Lf has shown its potential in various aspects of bone remodeling, signaling, fracture healing, peptide synthesis, and Lf delivery to bones. Further research in this area may lead to the development of new treatments for bone-related disorders.

## Author contributions

MT, Y-BH, G-YY, J-LL, and C-SH conducted literature search and wrote the manuscript. DT conceptualized the theme of the review and finalized the manuscript. DT takes responsibility for the integrity of the substance described in the review as a whole as ‘guarantor’. All authors contributed to the article and approved the submitted version.
